# Population genomics of the neotropical palm *Copernicia prunifera* (Miller) H. E. Moore: Implications for conservation

**DOI:** 10.1371/journal.pone.0276408

**Published:** 2022-11-03

**Authors:** Marcones Ferreira Costa, Jonathan Andre Morales-Marroquín, Carlos Eduardo de Araújo Batista, Alessandro Alves-Pereira, Fábio de Almeida Vieira, Maria Imaculada Zucchi

**Affiliations:** 1 *Campus* Amílcar Ferreira Sobral, Federal University of Piauí, Floriano, Piauí, Brazil; 2 Graduate Program in Genetics and Molecular Biology, Institute of Biology, State University of Campinas, São Paulo, Brazil; 3 Department of Genetics, “Luiz de Queiroz” College of Agriculture, University of São Paulo, Piracicaba, São Paulo, Brazil; 4 Academic Unit Specialized in Agricultural Sciences, Federal University of Rio Grande do Norte, Macaíba, Brazil; 5 Paulista Agency of Agrobusiness Technology, Piracicaba, São Paulo, Brazil; Institute for Biological Research, University of Belgrade, SERBIA

## Abstract

*Copernicia prunifera* (Miller) H. E. Moore is a palm tree native to Brazil. The products obtained from its leaf extracts are a source of income for local families and the agroindustry. Owing to the reduction of natural habitats and the absence of a sustainable management plan, the maintenance of the natural populations of this palm tree has been compromised. Therefore, this study aimed to evaluate the diversity and genetic structure of 14 *C*. *prunifera* populations using single nucleotide polymorphisms (SNPs) identified through genotyping-by-sequencing (GBS) to provide information that contributes to the conservation of this species. A total of 1,013 SNP markers were identified, of which 84 loci showed outlier behavior and may reflect responses to natural selection. Overall, the level of genomic diversity was compatible with the biological aspects of this species. The inbreeding coefficient (*f*) was negative for all populations, indicating excess heterozygotes. Most genetic variations occurred within populations (77.26%), and a positive correlation existed between genetic and geographic distances. The population structure evaluated through discriminant analysis of principal components (DAPC) revealed low genetic differentiation between populations. The results highlight the need for efforts to conserve *C*. *prunifera* as well as its distribution range to preserve its global genetic diversity and evolutionary potential.

## Introduction

Habitat reduction and deforestation resulting from human activities have had adverse effects on forest populations, contributing to high rates of species extinction, particularly in the Neotropical region [1]. With the exception of some natural areas, most tropical species occur in anthropogenic landscapes, where the previously continuous forest has now been reduced to smaller and isolated patches [2]. This modification of landscape composition and structure leads to habitat fragmentation, contributing to the loss of alleles, reduction of heterozygosity, and increase in inbreeding [3].

The palm tree *Copernicia prunifera* (Mill.) H. E. Moore (Arecaceae; subfamily: Coryphoideae), known as carnaúba, generally forms monodominant populations known as “carnaubais” [4]. The species has multiple inflorescences, which are made up of yellowish and hermaphroditic flowers [5]. Flowering is more intense between November and February, and the fruiting period is between January and March [6]. Fruits are likely dispersed by sanhaçu-do-coqueiro (*Tangara palmarum*) [5].

*C*. *prunifera* is endemic to the Caatinga biome [7], which is one of the largest seasonally dry tropical forest areas in South America [8]. The Caatinga is an exclusively Brazilian biome, covering an area of approximately 900,000 km^2^ in northeast Brazil. The climate in this region is characterized by a long dry season with irregular rainfall, representing a xeric, semi-deciduous shrubland and forest vegetation [9]. This palm tree also grows in the Restinga region, which contains vegetation of the coastal plain under marine influence and established on sandy soil composed of physiognomic variations from the beach towards the interior of the coastal plain [10,11]. Caatinga and Restinga are the major vegetation units in northeast Brazil [12].

Local populations use this species as a source of employment and income. Leaf extraction is responsible for sustaining several families during the period of drought that extends from July to December in northeast Brazil [13]. The fruits serve as food for animals, the stems can be used in the construction of houses, and the fasciculated roots have medicinal properties [14]. Due to its versatility and usefulness, this palm tree is known as “the tree of life” [15]. The main product of economic value obtained from this tree is carnaúba wax, which is extracted from young leaves and is of interest in the pharmaceutical and automotive industries [16]. Carnaúba populations suffer from intense exploitation because the method used to extract carnauba wax, which consists of practically removing all the leaves of the plant to obtain ceriferous powder [17].

**Fig 1:**
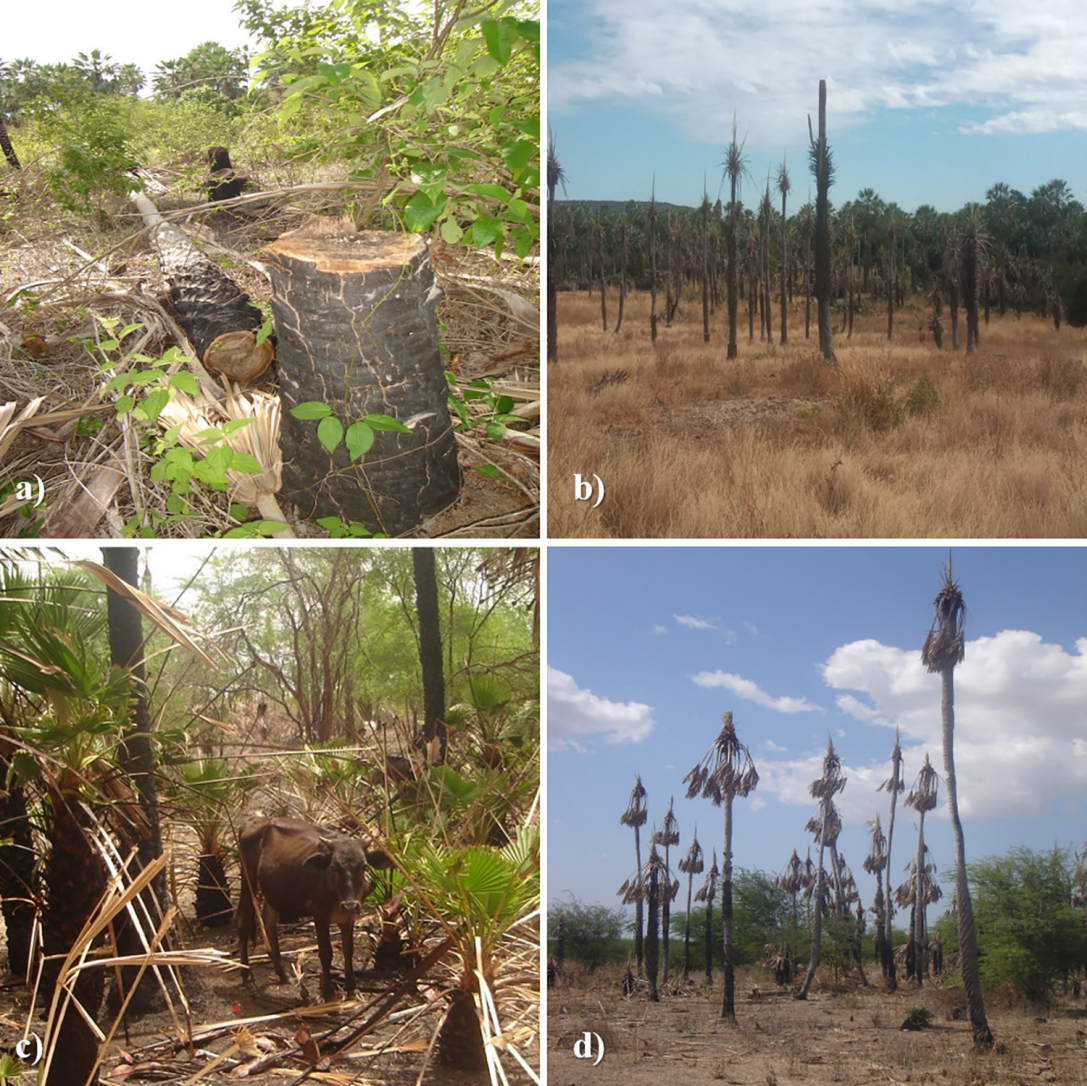
Signs of anthropization in the evaluated populations of *C*.*prunifera*. a) wood cutting; b) leaf extraction and cutting; c) cattle raising; and d) burns.

However, unsustainable harvesting practices and the absence of sustainable management programs pose major threats to the long-term survival of this palm tree species. *C*. *prunifera* populations show signs of intense exploitation with visible signs of anthropogenicity, such as fire, extraction and cutting of leaves, soil impacted by livestock, and low or absence of regeneration (Fig 1). In addition, anthropogenic disturbances in the last century, mainly due to deforestation, agricultural expansion, and modernization of agriculture, have led to a rapid decline in these populations [17].

The maintenance of genetic diversity is a powerful conservation strategy for preserving the adaptive potential of species in neotropical regions [18]. In addition to configuring the ability of species to adapt to various changing environments, genetic diversity is the driving force behind evolution and speciation. [19]. Consequently, maintenance of genetic diversity within populations ensures that the species can remain biologically active and adaptable to structural changes caused by anthropogenic actions [20,21].

The genetic diversity and structure of forest populations evaluated based on molecular markers is a widely used strategy in conservation genetics [22,23]. With the advent of next-generation sequencing, it is possible now to identify thousands of molecular markers of single nucleotide polymorphism (SNP) throughout the genome. This provides a genomic approach to evaluating genetic diversity [24]. A larger SNP sample size facilitates the identification of regions that show signs of selection and can serve as a starting point for the identification of adaptive differences between populations, which is fundamental for optimizing biological conservation efforts [25,26]. These markers enable the identification of outlier and neutral loci. Specifically, outlier loci show differentiated behavior regarding genetic variation and offer an opportunity to evaluate local adaptation patterns; neutral loci are similarly affected by the demographic and evolutionary history of populations [27].

Genetic diversity studies based on molecular markers of natural populations of *C*. *prunifera* in tropical areas such as Caatinga and Restinga are still scarce [17,28–30]. In addition, no studies on *C*. *prunifera* have applied next-generation sequencing technology for data acquisition in population genomics. Due to the importance of this neotropical palm tree for local communities and considering the rapid and recent increases in the exploitation of its populations, the present study employed next-generation sequencing to evaluate the genetic diversity and structure of 14 natural populations of *C*. *prunifera* in two environments (Caatinga and Restinga) in Brazil using SNP markers to provide information that can help in the design of efficient strategies for the conservation and sustainable use of this species.

## Material and methods

### Plant material and DNA extraction

**Fig 2:**
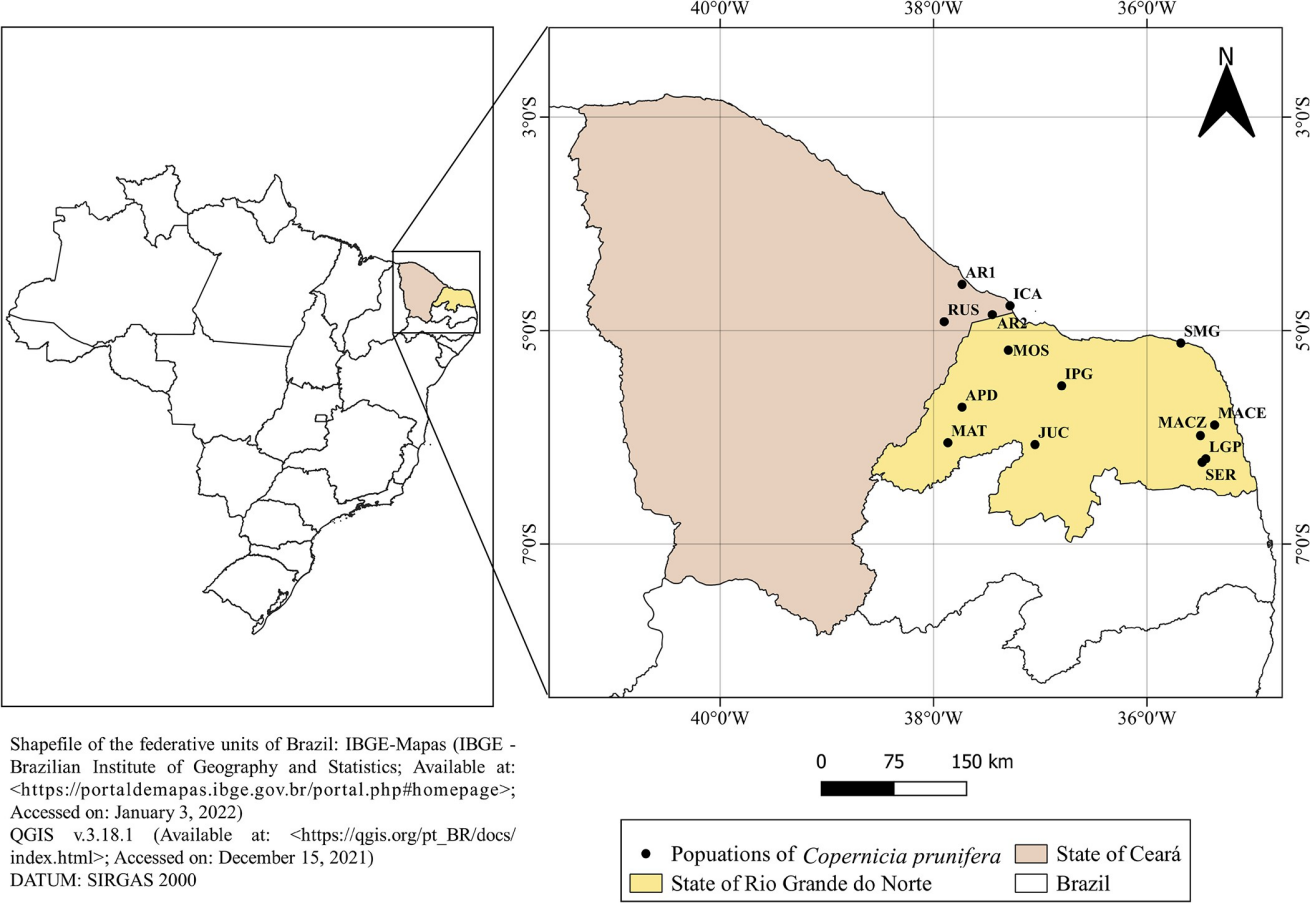
Map of the collection sites of *C*. *prunifera* populations in the states of Ceará and Rio Grande do Norte, Brazil. Distribution map of the evaluated populations was drawn using the software QGIS v3.18.1. (Open Access Geographic Information System, https://qgis.org/pt_BR/site). This figure is licensed under CC BY 4.0.

In the present study, 160 individual plants from 14 populations of *C*. *prunifera* were evaluated. Out of the samplings collected, 10 populations came from the Caatinga (RUS, LGP, SER, MACZ, MACE, JUC, APD, IPG, MOS, and MAT) and four from the Restinga (ICA, SMG, AR1, and AR2) regions in the states of Ceará and Rio Grande do Norte, Brazil (Fig 2 and S1 Table). The distance between the plants evaluated within the 14 populations was 15–20 m, with a minimum height of 6–10 m; regenerating and young plants were not collected. The IPG population is composed of a different type of carnaúba, known as “white carnaúba,” which is phenotypically distinct from the “common carnaúba” due to the presence of a light stipe, smaller fruits, and the absence of thorns in the petiole in addition to limited occurrence in the region [14].

Small pieces of leaves were cut using a tree trimmer, placed in plastic tubes containing 2 mL of hexadecyltrimethylammonium bromide (CTAB 2X), labeled, and stored in a freezer at -20°C until DNA extraction. This study was conducted according to the recommendations of the Brazilian Ministry of the Environment and registered in the National System of Management of Genetic Heritage and Associated Traditional Knowledge (SISGEN; *Sistema Nacional de Gestão do Patrimônio Genético e do Conhecimento Tradicional Associado*) with the number A411583.

Genomic DNA was extracted from the processed leaves according to the protocol described by Doyle and Doyle [31]. DNA quality was evaluated in a 1% agarose gel and stained with SYBR Safe™ (Life Technologies Corporation) for visualization under ultraviolet light, using lambda phage DNA of known concentrations as a reference. Quantification of the samples was performed using a Qubit 3.0 fluorometer with the dsDNA BRKitt (Life Technologies), and the DNA was standardized to a concentration of 30 ng.μl^-1^

### GBS library preparation and high-performance sequencing

To obtain SNPs, genomic libraries were developed using the genotyping-by-sequencing technique (GBS) with two restriction enzymes, according to the protocol described by Poland et al. [32] with modifications. First, 7 μl of genomic DNA from each sample was digested at 37°C for 12 h using the restriction enzymes *NsiI* and *MspI*. Subsequently, 0.02 μM barcode-specific adapters for Illumina technology were ligated to the ends of the digested fragments. Binding reaction was performed at 22°C for 2 h, 65°C for 20 min, 10°C indefinitely. After the adapters were ligated, the samples were purified using a QIAquick PCR Kit (Qiagen). The library was enriched by PCR (Polymerase Chain Reaction) using the following amplification program: 95°C for 30 s, followed by 16 cycles of 95°C for 10 s, 62°C for 20 s, and 72°C for 30 s, and ending at 72°C for 5 min. Finally, the library was purified using a QIAgen^®^ QIAquick PCR Purification Kit. The Agilent DNA 12000 kit and Agilent^®^ 2100 Bioanalyzer System were used to verify the average size of the DNA fragments. Sequencing was performed using the Illumina^®^ HiSeq 2500 Mid Output Kit v4 (50 cycles) (Illumina Inc., San Diego, CA, USA) in a single-end configuration.

### Identification of SNPs

The identification of SNPs was performed using Stacks software v.1.42 [33,34]. The first step comprised filtering and demultiplexing with the *process_ radtag* module. In the absence of a reference genome for *C*. *prunifera*, the *DeNovo* Stacks pipeline was used, starting with the *ustacks* module to identify putatively homologous read stacks (putative loci). This step was performed for each sample separately using the following parameters: minimum stack depth (-m = 3) and maximum distance between stacks (-M = 2). The loci of each sample were grouped into a catalog using the *cstacks* module, allowing a maximum distance of two nucleotides (-n 2) between the loci of each sample. Loci with lower probability values (—lnl_lim -10) were eliminated using the *rxstacks* correction module. Finally, the *population* module was used to filter the SNP markers using the following parameters: only one marker per sequenced tag, frequency of least frequent allele (MAF ≥ 0.01), minimum stack depth 3X, and minimum occurrence in 75% of saplings in each population.

### Loci determination under selection

Two complementary tests were performed, pcadapt and fsthet, were performed to detect outlier loci (hypothetically under selection). The pcadapt method [35] was used to identify loci associated with the genetic structure revealed by a principal component analysis (PCA), that is, without any underlying genetic model. The analysis was performed using the pcadapt package [35] on the R platform [36] by retaining the first eight principal components of the PCA and considering the loci with q-values ≤ 0.1 as outlier SNPs. The fsthet method [37] was used to identify loci with F_ST_ values that were excessively high or low compared with what was expected under neutrality. The analysis was performed using the fsthet package [37] on the R platform [36] by considering the loci below or above the 95% confidence intervals constructed with 1000 bootstraps for the expected relationship between H_E_ and F_ST_ as outlier SNPs. This test was performed by considering the estimates of F_ST_ in two different scenarios: i) comparing the Restinga and Caatinga populations and ii) comparing the samples of the white morphotype with those of conventional morphotype. The final set of SNP markers hypothetically under selection consisted of loci identified as outliers in at least two of the three tests performed. Thus, the outlier SNP loci may reflect the action of selection on different types of vegetation.

Sequences containing outlier SNPs were searched with the BLASTX tool against the genomic data set of the National Center for Biotechnology Information (NCBI) using blast2go [38]. This analysis was performed to identify the similarities between the protein-coding data deposited in the NCBI database and the loci with outlier SNPs identified. For sequences with significant BLASTX hits, the functional annotation associated with characterized and/or described coding sequences was performed using the gene ontology system (GO terms). GO terms summarize information on cellular components, molecular functions, and biological processes in which the gene products are involved.

### Population genomic analyses

Genetic diversity was estimated based on the number of alleles (A), number of private alleles (A*p*), observed heterozygosity (H_*O*_), and expected heterozygosity (H_*E*_). Inbreeding coefficients (*f*) were also estimated, and their confidence intervals were obtained using 1000 bootstraps. Estimates of diversity and inbreeding were obtained using the diveRsity [39] and PoPPr [40] packages of the R software [36]. The distribution of genetic variation within and between populations of *C*. *prunifera* was evaluated using analysis of molecular variance (AMOVA), and its significance was tested with 10,000 permutations using the PoPPr program [40].

Genetic differentiation was estimated using pairwise *F*_*ST*_ values with confidence intervals of 1000 bootstraps, using the diveRsity package [39] of R software [36]. The population structure was evaluated using discriminant analysis of principal components (DAPC) with Adegenet [41,42] for R software [36]. This analysis was performed for neutral loci, and *priori* groups were defined from the 14 sampling sites. DAPC does not presuppose the underlying population genetic processes (e.g., binding equilibrium and Hardy–Weinberg equilibrium) common to other methods used to detect population structure, and as it is based on principal component analysis, this method can analyze genomic datasets relatively efficiently [43].

Genetic relationships and divergence between individuals were investigated by constructing a dendrogram generated based on the distance of Nei using the neighbor-joining method [44]. The final dendrograms were formatted using MEGA version 7 [45].

## Results

### Identification of SNPs and determination of loci under selection

**Fig 3:**
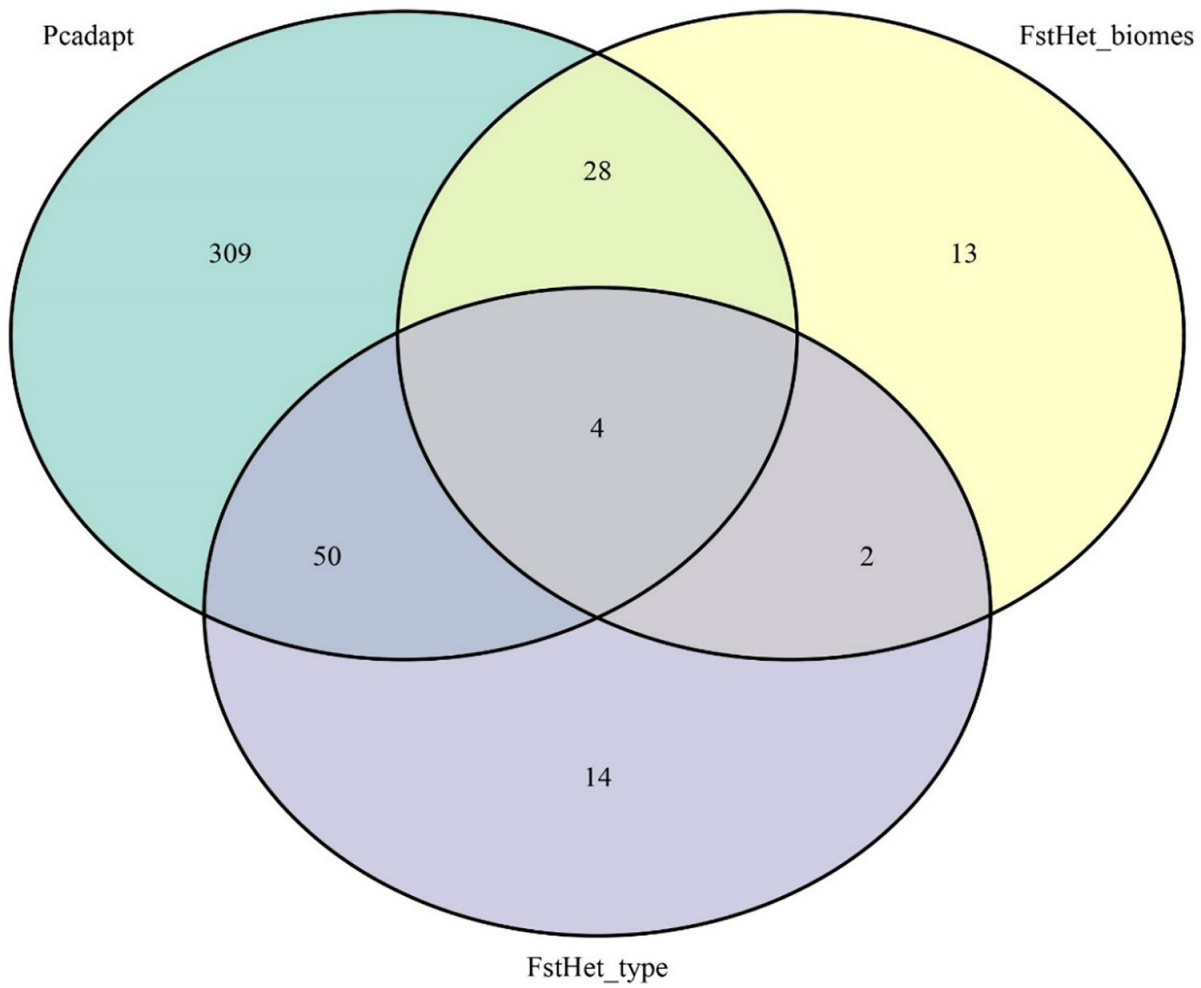
Venn diagram with the number of outlier loci detected for the fsthet and Pcadapt tests with the overlap between them.

Sequencing of the genomic libraries resulted in 566,922,165 reads, and after quality control, the total number of reads retained was 397,047,980. In total, 1,013 SNPs (average depth of 21X) were identified. A total of 391 outlier SNPs were identified using the pcadapt method, 70 using the fsthet method to compare morphotypes, and 47 using the fsthet method to compare vegetation types (Fig 3). Of these, 84 SNP markers were identified in at least two of the three tests and were considered hypothetically under selection, whereas the other 929 markers were considered as neutral loci. Among the outlier loci, 55 were putatively under positive selection and 29 were putatively under balancing selection. Only six outlier loci were found in the sequences similar to the annotated proteins (S2 Table). Considering the results of the GO terms, the most frequent annotations for these proteins were the molecular functions of “binding” and “catalytic activity,” and the biological process of cell metabolism (S1 Fig).

### Population genomic analyses

The genomic diversity estimates were based on 929 neutral SNP markers. The number of alleles (A) ranged from 1,059 to 1,497. The IPG population (white carnaúba) had the lowest number of alleles, probably because of the small sample size of the population. Expected heterozygosity (H_*E*_) ranged from 0.201 to 0.265 (Table 1). The APD population had higher genetic diversity (H_*E*_ = 0.265) and the highest number of private alleles (Ap = 40) compared with that of the other populations. The inbreeding coefficients (*f*) were similar and negative for all populations, indicating an excess of heterozygotes.

**Table 1:** Estimates of genomic diversity and inbreeding based on 929 neutral SNP markers for populations of *C*. *prunifera*.

Population	N	A	AP	H_*O*_	H_*E*_	*f*	*f* (95% CI)
**ICA**	7	1439	2	0.375	0.257	-0.367	(-0.473 - -0.308)
**AR1**	17	1497	35	0.387	0.260	-0.391	(-0.439 - -0.357)
**AR2**	11	1484	3	0.388	0.258	-0.454	(-0.526 - -0.394)
**RUS**	21	1495	27	0.383	0.260	-0.287	(-0.556 - -0.326)
**SMG**	10	1353	16	0.370	0.235	-0.272	(-0.34 - -0.211)
**LGP**	6	1059	5	0.378	0.229	-0.553	(-0,642 - -0.473)
**SER**	10	1235	6	0.381	0.246	-0.438	(-0,556 - -0.326)
**MACZ**	10	1462	9	0.387	0.259	-0.405	(-0,461- -0.365)
**MACE**	14	1227	7	0.369	0.201	-0.102	(-0,126- -0.081)
**JUC**	12	1466	3	0.376	0.247	-0.447	(-0,564 - -0.38)
**APD**	12	1317	40	0.372	0.265	-0.238	(-0,383 - -0.0127)
**IPG**	5	1249	1	0.375	0.224	-0.123	(-0,209 - -0.049)
**MOS**	18	1404	28	0.380	0.255	-0.112	(-0.174 - -0.065)
**MAT**	7	1361	8	0.376	0.237	-0.353	(-0.437- -0.306)

Number of individuals (N); total number of alleles (A); number of private alleles (Ap); observed heterozygosity (H_*O*_); expected heterozygosity (H_*E*_); inbreeding coefficient (*f*); *f* (CI of 95%) = lower and upper confidence interval of 95% of the inbreeding coefficients.

F_*ST*_ values 0–0.05 and 0.05–0.15 indicate low and moderate genetic differentiation, respectively, whereas values > 0.15 indicate high differentiation (Hartl, Clark 1997). In the present study, F_*ST*_ estimates suggested low to high genetic differentiation between populations of *C*. *prunifera* (Table 2). In general, there was a greater differentiation between the population from MACE and those from the other sites (F_*ST*_ ranged from 0.118 to 0.20). In addition, the SMG and IPG populations showed moderate levels of differentiation. A low genetic structure was observed for the populations from LGP and SER (0.18) and AR1 and AR2 (0.19), suggesting a genetic flow between these localities.

**Table 2:** Estimates of pairwise F_*ST*_ between populations of *C*. *prunifera* (lower diagonals). Upper diagonals contain the lower and upper limits of the confidence interval.

	IPG	APD	AR2	JUC	LGP	MACE	MACZ	MAT	MOS	RUS	SER	AR1	ICA	SMG
**IPG**		(0.05–0.14)	(0.02–0.11)	(0.05–0.20)	(0.05–0.17)	(0.17–0.27)	(0.03–0.12)	(0.06–0.19)	(0.04–0.14)	(0.03–0.13)	(0.05–0.16)	(0.04–0.13)	(0.02–0.13)	(0.05–0.16)
**APD**	0.092		(0.01–0.07)	(0.01–0.12)	(0.03–0.12)	(0.12–0.19)	(0.02–0.08)	(0.06–0.13)	(0.02–0.08)	(0.01–0.06)	(0.02–0.09)	(0.02–0.07)	(0.00–0.09)	(0.07–0.14)
**AR2**	0.053	0.036		(0.02–0.11)	(0.03–0.10)	(0.13–0.20)	(0.01–0.05)	(0.03–0.10)	(0.01–0.06)	(0.00–0.04)	(0.02–0.08)	(0.00–0.04)	(-0.03–0.03)	(0.04–0.09)
**JUC**	0.109	0.062	0.057		(0.03–0.15)	(0.11–0.18)	(0.01–0.09)	(0.01–0.15)	(0.05–0.14)	(0.03–0.11)	(0.02–0.10)	(0.03–0.11)	(0.02–0.12)	(0.06–0.14)
**LGP**	0.096	0.064	0.054	0.072		(0.10–0.16)	(0.02–0.09)	(0.06–0.15)	(0.06–0.13)	(0.04–0.09)	(-0.01–0.07)	(0.02–0.08)	(0.01–0.11)	(0.05–0.13)
**MACE**	0.210	0.150	0.162	0.144	0.123		(0.09–0.15)	(0.17–0.23)	(0.15–0.21)	(0.13–0.17)	(0.09–0.15)	(0.12–0.16)	(0.14–0.21)	(0.12–0.17)
**MACZ**	0.063	0.046	0.027	0.042	0.051	0.118		(0.04–0.10)	(0.03–0.09)	(0.01–0.05)	(0.02–0.08)	(0.02–0.06)	(0.00–0.07)	(0.03–0.08)
**MAT**	0.113	0.087	0.058	0.073	0.094	0.195	0.063		(0.06–0.12)	(0.04–0.09)	(0.06–0.12)	(0.04–0.10)	(0.03–0.10)	(0.08–0.15)
**MOS**	0.077	0.047	0.032	0.084	0.086	0.172	0.050	0.084		(0.02–0.07)	(0.05–0.10)	(0.03–0.07)	(0.00–0.07)	(0.06–0.11)
**RUS**	0.068	0.033	0.016	0.060	0.057	0.148	0.030	0.054	0.041		(0.04–0.09)	(0.01–0.04)	(0.00–0.05)	(0.05–0.11)
**SER**	0.094	0.053	0.045	0.054	0.018	0.118	0.046	0.080	0.069	0.063		(0.03–0.07)	(0.02–0.08)	(0.05–0.10)
**AR1**	0.074	0.041	0.019	0.062	0.043	0.138	0.034	0.064	0.045	0.019	0.048		(0.01–0.07)	(0.05–0.10)
**ICA**	0.060	0.036	-0.003	0.058	0.050	0.171	0.028	0.055	0.032	0.022	0.044	0.029		(0.05–0.11)
**SMG**	0.090	0.101	0.064	0.098	0.081	0.142	0.048	0.102	0.079	0.077	0.070	0.073	0.072	

**Fig 4:**
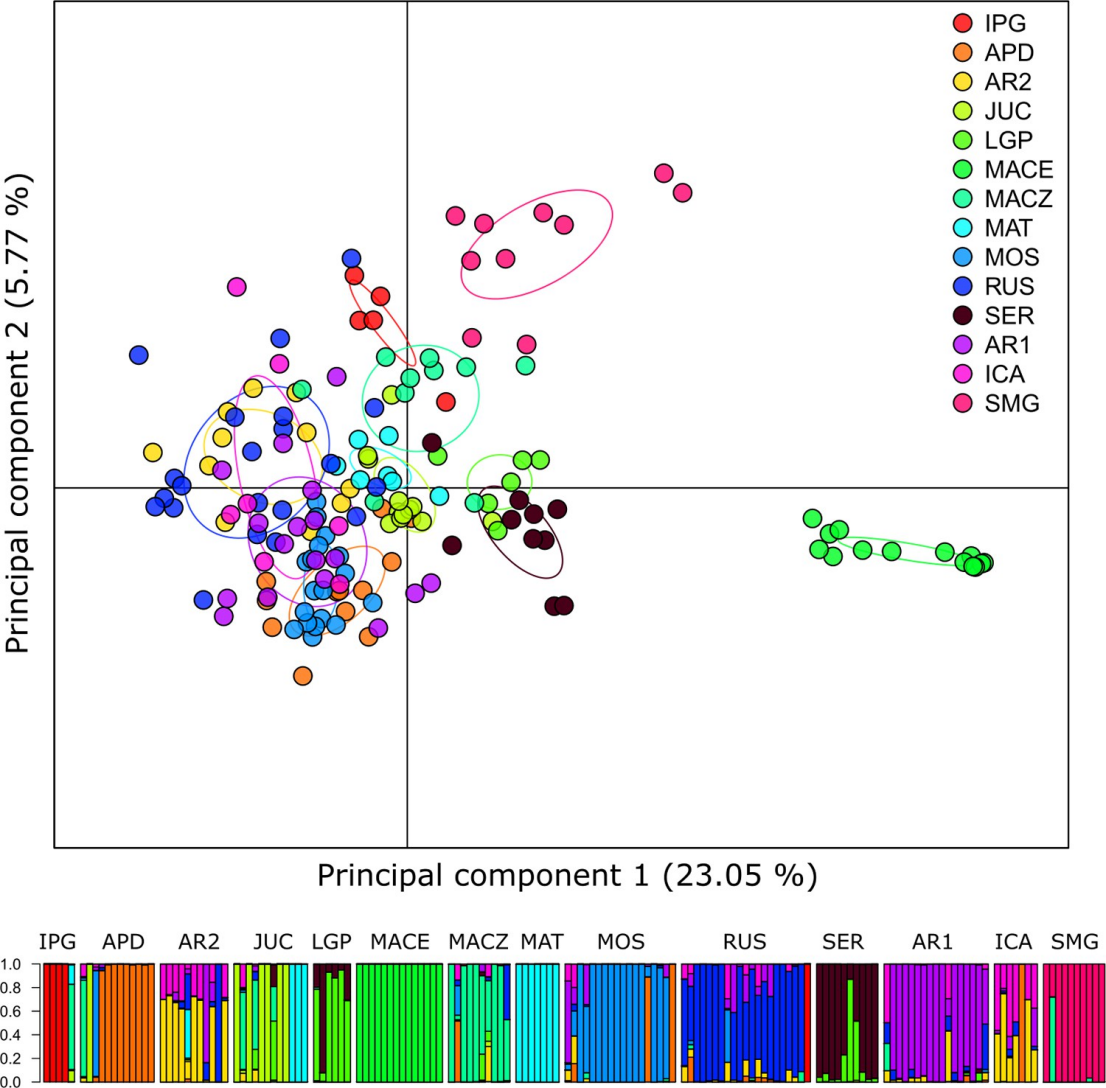
a) Discriminant analysis of principal components (DAPC) representing the genetic structure of *C*. *prunifera* populations based on 929 SNPs. b) Bar graph representing the coefficients of DAPC, where each bar delimits one individual.

The low genetic divergence suggested by the pairwise F_*ST*_ was also observed in DAPC, which retained 28.7% of the total variation in the first two principal components (Fig 4). This analysis also showed greater genetic differentiation of the population from MACE in comparison with that of others in addition to pointing out an overlap between individuals from almost all populations, especially AR1, AR2, and RUS.

Analysis of molecular variance (AMOVA) indicated that most of the variation was found within populations (77.26%), and the genetic differentiation between populations was high and significant (φ = 0.227) (Table 3). The Mantel test revealed a positive and significant correlation between the geographical and genetic distances based on the F_*ST*_ values (r = 0.0612; p = 0.002).

**Table 3:** Analysis of molecular variance (AMOVA) based on 929 neutral SNP markers for fourteen natural populations of *C*. *prunifera*.

	Degrees of freedom	Sum of the squares	Mean square	Variance	Percentage	*F*_*ST*_ global (φ)	*p*-value
**Between the populations**	13	3307.963	254.45867	17.32471	22.7366	0.227366	0.00005
**Within the population**	146	8595.417	58.87272	58.87272	77.2634		
**Total**	159	11903.38	74.86402	76.19743			

**Fig 5:**
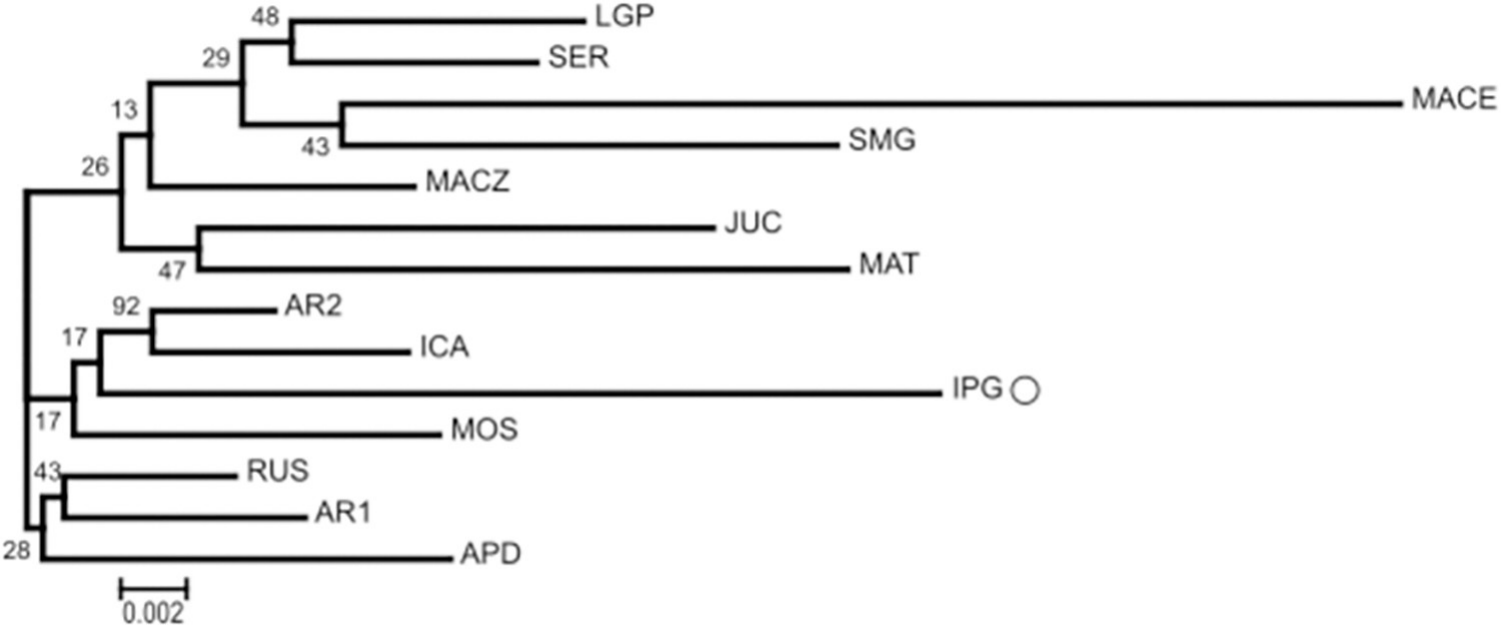
Dendrogram obtained by the neighbor-joining method based on SNP markers for the 14 populations of *C*. *prunifera*.

According to the dendrogram (Fig 5), the MACE population was the most genetically distant, corroborating the results observed in the DAPC population. Individual saplings from the LGP and SER populations exhibited similar levels of genetic similarity. In addition, there was a clear distinction among the three groups: the first group was formed by the populations from LGP, SER, MACE, SMG, MACZ, JUC, and MAT; the second group consisted of AR2, ICA, IPG (white carnaúba), and MOS; and the third group consisted of RUS, AR1, and APD. Populations from AR2 and ICA had the highest bootstrap value, which indicates a statistically well-supported grouping.

When the genetic diversity and structure of the *C*. *prunifera* populations were evaluated based on the type of vegetation (Caatinga and Restinga), similar levels of genetic diversity were observed (Table 4).

**Table 4:** Estimates of genomic diversity and inbreeding based on 929 neutral SNP markers for populations of *C*. *prunifera*, considering the different types of vegetation (Caatinga and Restinga) and morphotypes (common carnaúba and white carnaúba).

Population	N	A	AP	H_*O*_	H_*E*_	*f*	*f* (95% CI)
**Caatinga**	126	1802	253	0.379	0.270	-0.400	(-0.417 - -0.382)
**Restinga**	34	1603	54	0.380	0.266	-0.432	(-0.468 - -0.397)
**Common carnaúba**	155	1855	607	0.348	0.271	-0.400	(-0.417 - -0.384)
**White carnaúba**	5	1249	1	0.345	0.224	-0.123	(-0.209 - -0.048)

Number of individuals (N); total number of alleles (A); number of private alleles (Ap); observed heterozygosity (H_*O*_); expected heterozygosity (H_*E*_); inbreeding coefficient (*f*); *f* (CI of 95%) = lower and upper confidence interval of 95% of the inbreeding coefficients.

The populations from the Caatinga had the largest number of private alleles (Ap = 253) compared to the Restinga populations and this result is probably associated with the sample size. The inbreeding coefficients (*f*) are both similar and negative. In addition, the F_*ST*_ estimates suggested low genetic differentiation between the Caatinga and Restinga populations (F_*ST*_ *=* 0.008). When considering only two morphotypes of *C*. *prunifera* (white carnaúba and common carnaúba), similarities were observed in the estimates of diversity in addition to low genetic differentiation (F_*ST*_ *=* 0.008) (Table 4). Analysis of molecular variance (AMOVA) among the vegetation types (Caatinga and Restinga) produced small genetic differentiation (φ   =  0.024). It revealed 97.522% of the genetic variation within the vegetation types whereas, 2.478% of the total genetic variation was observed between types of vegetation (Table 5).

**Table 5:** Molecular analysis of variance (AMOVA) considering the Caatinga and Restinga for the populations of *C*.*prunifera*.

	Degrees of freedom	Sum of the squares	Mean square	Variance	Percentage	*F*_*ST*_ global (φ)	*p*-value
**Between types of vegetation**	1	175.234	175.23395	1.886185	2.478	0.02478074	0.00005
**Within types of vegetation**	158	11728.146	74.22877	74.22877	97.522		
**Total**	159	11903.38	74.86402	76.114954			

## Discussion

### Loci putatively under selection

The large number of SNP markers obtained in this study allowed for the identification of loci with deviations from the expected neutral behavior, which are putatively under selection (outlier loci). The identification of outlier loci is an important step in understanding local adaptation and evaluating the evolutionary potential of a species [46]. The palm tree *C*. *prunifera* has no annotated reference genome, and probably for this reason, most sequences with outlier loci are similar to uncharacterized proteins. Regarding the results obtained from the annotation, most loci are associated with genes involved in metabolic processes, which have been regularly found under selection in a variety of organisms because the gene functionality correlates with environmental stressors [47].

Interestingly, some annotated loci were associated with genes of transposable elements (S2 Table). According to Gogvadze and Buzdin [48], transposable elements promote changes in the genome, which is an important evolutionary mechanism for the adaptation of organisms to changes in environmental conditions. This is expected in *C*. *prunifera* because the palm trees grow in different environments such as seasonally flooded areas in the semi-arid region [49]. In addition, outlier loci may be associated with environmental differences in the collection sites, especially as sampling areas are scattered over the Restinga and Caatinga.

It is important to highlight that the analyses performed in this study are unable to indicate associations between genomic and functional variation; therefore, it is not possible to associate generic molecular functions or biological processes with any adaptive traits involved in the diversification of the evaluated populations. Therefore, studies with larger sample sizes with better representation of the different geographical habitats are needed to generate information on the evolution and diversification of *C*. *prunifera*. Small sample sizes belonging to populations with relatively small geographical distances, which enable gene flow to quickly spread new adaptations to surrounding areas, reduce the capacity to detect recent evolutionary changes [50]. However, the identified outlier loci can be used as candidates in association mapping studies. Thus, integrative approaches of association genetics, genome-wide scans, and measurements of phenotype selection are necessary to understand the adaptive nature of a given allele [51].

### Genetic diversity, inbreeding, and structure

Genetic diversity is one of the three classes of biodiversity recognized as a global conservation priority and plays a decisive role in conservation efforts. Genetic diversity has a substantial effect on both individual fitness and the adaptive capacity of the population, playing a vital role in maintaining the capacity of species to withstand various biotic and abiotic stressors and evolve under altered environmental conditions [52]. The present study provides the first estimates based on SNPs for genetic diversity in *C*. *prunifera*. The GBS approach used in this study produced a large number of SNP loci for the genomic evaluation of this palm tree without the need for a reference genome. This has resulted in robust estimates of diversity and patterns of genetic structure.

The results of genetic diversity and population structure were similar based on the results of the analysis according to population (among the 14 localities), type of vegetation (Caatinga and Restinga), and morphotype (common carnaúba and white carnaúba). In all situations, the populations showed a negative *f* value, suggesting limited inbreeding with reduced self-pollination capacity under environmental conditions. Therefore, individual plants are less related than expected under conditions of random mating. Genetic diversity and population structure is influenced by biological characteristics of the species, including the mating system [53]. Therefore, the reproductive biology of this species may explain the observed patterns of genetic variation. The mating system of *C*. *prunifera* is mixed and preferably allogamous [5], which favors the crossing between unrelated individuals. Thus, inbreeding coefficients are reduced, and the maintenance of genetic diversity within populations is ensured.

Although the evaluated populations were susceptible to anthropogenic threats, it is possible that they had high genetic diversity (H_*E*_). High levels of genetic diversity led to an increase in long-term survival of a species; therefore, a strong positive correlation exists between heterozygosity and population fitness, which is important for populations to adapt to new environmental conditions [54]. This high level of diversity is expected in forest species that are largely not domesticated as a result of local adaptation and neutral evolutionary processes in heterogeneous environments [22].

The identification of private alleles is useful for genetic conservation [55]. In the present study, the populations from APD (Ap = 40), AR1 (Ap = 35), and MOS (Ap = 28) had the highest number of private alleles and diversity was not found in the other localities; therefore, these populations deserve special management because the levels of private alleles are indicative of individual fitness and explain the evolutionary potential of populations and their ability to adapt to the adverse environmental conditions [21]. Therefore, this information can be used to increase the genetic representation in germplasm banks. and to convey the need to explore seed collection *in situ* to ensure future replacement.

Genetic variation in plant species is strongly affected by several historical and demographic factors, including geographic distribution, life form, and population size [56]. The results of AMOVA showed that most of the genetic diversity was found within *C*. *prunifera* populations (Table 3). Similarly, Santos et al. [17] analyzed the genetic differentiation of this palm tree in the northeast region of Brazil and found that 62.86% of molecular variance was accounted for by differences within populations. These results agree with those of different studies conducted on forest species that reproduce by allogamy, seeing as these species have maintained most of their genetic variability within populations [57].

Genetic structure analyses indicated that the 14 collection sites did not belong to a single homogeneous population, and the geographically closest populations showed low values of pairwise F_*ST*_ and overlap in the DAPC. Greater genetic similarity was found between the populations from LGP and SER and between AR1 and AR2. In addition, low genetic differentiation was observed when the populations were evaluated according to vegetation type and morphotype.

The low level of global genetic differentiation found between the populations studied here (supported by F_*ST*_, cluster analysis, and DAPC) and the higher proportion of genetic diversity within populations with only fewer partitions between them could result from the combined effect of different factors, such as cross rate, reproductive system, and high genetic flow rate in this species. The Mantel test corroborates this result. Since geographically close populations tend to be genetically similar, this indicates a pattern of isolation by distance. However, the MACE population had the lowest level of diversity (H_*E*_ = 0.201) and the highest degree of structuring, being the most genetically divergent population compared with the others. This differentiation was supported by the F_*ST*_ value, which is an indirect estimator of the population connectivity between subpopulations (Table 2). The population from MACE corresponds to a small population with the lowest number of plants in an area of approximately 0.9 hectares, and spatially isolated from other populations. Furthermore, anthropogenic factors are observed in higher intensity in this population, this fact is probably related to the intense exploitation of carnauba wax in this area [14]. These factors can lead to a reduction in genetic diversity within population, likely as a result of genetic drift [58]. Santos et al. [17], using ISSR markers, also indicated that MACE population has a high differentiation, and genetic discontinuities were observed between this population and the others, with indications of a recent genetic bottleneck. Therefore, the impact of human activities may have contributed to the levels of genetic differentiation observed in the MACE population.

### Implications for conservation

Conservation genomics is an extension of conservation genetics that seeks to apply genomic techniques to the practical management of natural populations [59]. In this context, evaluations of genetic variations in the entire genome are powerful approaches to gain an understanding of the processes that lead to molecular diversification and inform effective management and conservation strategies [60]. However, application in real-time has been slow and a persistent gap exists between theory and practice.

In Brazil, the legislation that guides forest management does not clearly describe the importance of genetic evaluation within natural populations; therefore, information that seeks to associate genetic data with the formulation of sustainable management plans is unfortunately not mentioned [61]. Although *C*. *prunifera* is not listed as an endangered species, the expansion of agricultural activities over time has contributed to a reduction in its natural population [17]. Therefore, conservation measures are necessary to minimize the additional loss of alleles and to ensure the maintenance of genetic resources.

Conserving genetic diversity within a population should be the cornerstone of any conservation strategy aimed at ensuring the long-term persistence of species and habitats [62]. *In situ* and *ex situ* conservation strategies are considered promising alternatives for the conservation of forest genetic resources (FGR) and aim to maintain the genetic diversity of species over time, preserving the evolutionary processes and adaptive potential of populations [63]. Although *ex situ* approaches have the potential to conserve much of the biological diversity, they do have a limitation of being more suited and efficient for conservation in plants that have orthodox seeds. Therefore, *in situ* conservation is recommended for *C*. *prunifera* because this species contains recalcitrant seeds [64]. However, active management, including the establishment of *in vivo* seed banks and the promotion of natural regeneration, can prevent the decrease of population size, loss of genetic variability, and ensure long-term conservation [17].

The high genetic diversity observed in the evaluated populations of *C*. *prunifera* indicates the need for large areas of land dedicated to *in situ* conservation for capturing the existing genetic diversity of these populations. F_ST_ values estimated in the present study could help in recommending the optimal number of populations for sampling, including populations that had the highest estimates of diversity and the largest number of private alleles.

*C*. *prunifera* exploitation is an important source of employment and income for local communities in the semi-arid region of Brazil. In this context, the rational management of palm tree products should be a principal strategy in the efforts to conserve the natural habitats of the species. Another strategy aimed at conservation and sustainable use would be the development of a community and family forest management (CFFM) plan [65], which consists of the planning and management of actions and appropriate techniques for the sustainable use of forest resources aimed at traditional communities and family farmers [66].

In addition, practical measures aimed at successful plant regeneration, such as the pause of extractive activity during reproductive periods and the introduction of rotation cycles for leaf harvesting in the explored areas, need to be implemented for the sustainable management of carnaúba. However, the current social and economic conditions of workers employed in the activity of extraction and production of carnaúba wax must be considered. Workers in poorer areas need to be provided additional support, including investments, to maintain the balance between socioeconomic demand and conservation, which would pave the way to a more sustainable supply of resources while reducing the pressure of uncontrolled harvesting.

A third approach would be to preserve populations and divergent genetic groups identified in this study throughout their geographic distribution range through effective long-term genetic and ecological monitoring, stimulating the development of ecological corridors between fragments and natural forests, and avoiding the reduction of genetic variability. In addition, interdisciplinary programs that study different aspects of *C*. *prunifera* populations (e.g., habitat quality, impact of extractive activity on individuals, and genetic diversity) throughout their distribution would be fundamental for the successful implementation of species conservation management.

## Supporting information

S1 Fig Genetic ontology assignment graph (GO). GO Annotations are summarized into three main categories: cellular location, biological process and molecular function for carnaúba (*Copernicia prunifera*).
(DOCX)

S1 Table Collection sites of the evaluated populations of Copernicia prunifera. (DOCX)

S2 Table Similarity with proteins and Gene Ontology classifications obtained in blast2go for outlier SNPs putatively under selection in carnaúba (Copernicia prunifera). *H*_*E*_ = expected heterozygosity of the locus; *F*_*ST*_ = genetic divergence among groups of accessions estimated based on the locus; e-value = number of hits expected by chance (E = x 10); Sim (%) = BLASTX percentage of similarity between SNP tags and annotated proteins.
(DOCX)
