# Supplementary material for: Population genomics of the neotropical palm Copernicia prunifera (Miller) H. E. Moore: Implications for conservation
Source: PLoS One. 2022 Nov 3;17(11):e0276408. doi: 10.1371/journal.pone.0276408 (PMC9632875; doi:10.1371/journal.pone.0276408)
Supplement: S1 Fig — GO Annotations are summarized into three main categories: cellular location, biological process and molecular function for carnaúba (Copernicia prunifera). (DOCX) [file pone.0276408.s001.docx]

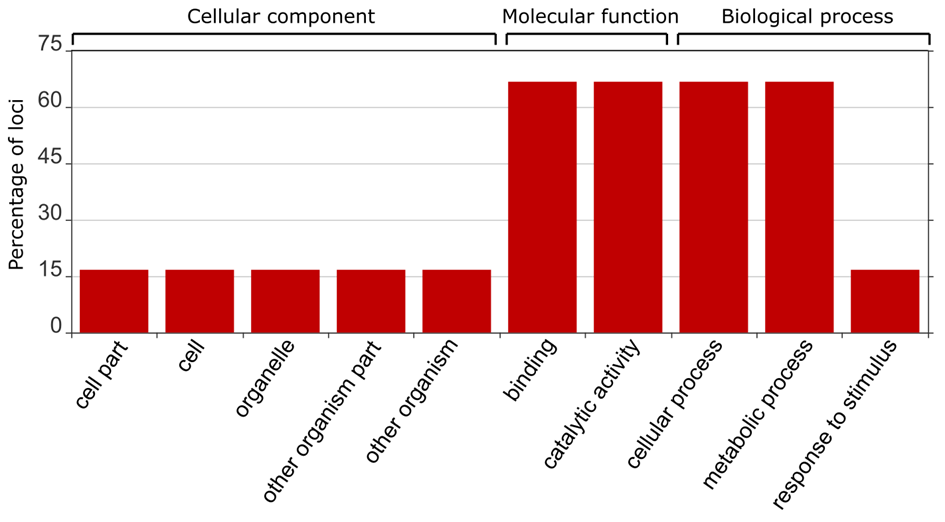


**S1 Fig. Genetic ontology assignment graph (GO). GO Annotations are summarized into three main categories: cellular location, biological process and molecular function for carnaúba (*Copernicia prunifera*)**
