# Supplementary material for: Population genomics of the neotropical palm Copernicia prunifera (Miller) H. E. Moore: Implications for conservation
Source: PLoS One. 2022 Nov 3;17(11):e0276408. doi: 10.1371/journal.pone.0276408 (PMC9632875; doi:10.1371/journal.pone.0276408)
Supplement: S1 Table — (DOCX) [file pone.0276408.s002.docx]

**S1 Table. Collection sites of the evaluated populations of *Copernicia prunifera***

| **Population** | **Initials** | **Stade** | **Latitude** | **Longitude** | **Biome** |
| --- | --- | --- | --- | --- | --- |
| Icapuí | ICA | Ceará | 4.766 | 37.283 | Restinga |
| Aracati 1 | AR1 | Ceará | 4.566 | 37.733 | Restinga |
| Aracati 2 | AR2 | Ceará | 4.85 | 37.45 | Restinga |
| Russas | RUS | Ceará | 4.916 | 37.9 | Caatinga |
| São Miguel do Gostoso | SMG | Rio Grande do Norte | 5.116 | 35.683 | Restinga |
| Lagoa de Pedras | LGP | Rio Grande do Norte | 6.2 | 35.45 | Caatinga |
| Serrinha | SER | Rio Grande do Norte | 6.233 | 35.483 | Caatinga |
| Macaíba (Zumbi) | MACZ | Rio Grande do Norte | 5.983 | 35.5 | Caatinga |
| Macaíba (EAJ) | MACE | Rio Grande do Norte | 5.883 | 35.366 | Caatinga |
| Jucurutu | JUC | Rio Grande do Norte | 6.066 | 37.05 | Caatinga |
| Apodi | APD | Rio Grande do Norte | 5.716 | 37.733 | Caatinga |
| Ipanguaçu | IPG | Rio Grande do Norte | 5.516 | 36.8 | Caatinga |
| Mossoró | MOS | Rio Grande do Norte | 5.183 | 37.3 | Caatinga |
| Martins | MAT | Rio Grande do Norte | 6.05 | 37.866 | Caatinga |
