# Supplementary material for: Population genomics of the neotropical palm Copernicia prunifera (Miller) H. E. Moore: Implications for conservation
Source: PLoS One. 2022 Nov 3;17(11):e0276408. doi: 10.1371/journal.pone.0276408 (PMC9632875; doi:10.1371/journal.pone.0276408)
Supplement: S2 Table — HE = expected heterozygosity of the locus; FST = genetic divergence among groups of accessions estimated based on the locus; e-value = number of hits expected by chance (E = x 10); Sim (%) = BLASTX percentage of similarity between SNP tags and annotated proteins. (DOCX) [file pone.0276408.s003.docx]

**S2 Table. Similarity with proteins and Gene Ontology classifications obtained in blast2go for outlier SNPs putatively under selection in carnaúba (*Copernicia prunifera*). *H_E_* = expected heterozygosity of the locus; *F_ST_* = genetic divergence among groups of accessions estimated based on the locus; e-value = number of hits expected by chance (E = x 10); Sim (%) = BLASTX percentage of similarity between SNP tags and annotated proteins.**

| **Locus** | ***H_E_*** | ***F_ST_*** | **e-value** | **Sim (%)** | **Molecular function** | **Biological process** |
| --- | --- | --- | --- | --- | --- | --- |
| 178120 | 0.495 | 0.114 | 3.41E-10 | 96.15 | Nucleic acid binding | DNA Biosynthesis |
| 113155 | 0.322 | 0.253 | 5.94 E-07 | 79.5 | Retrotransposon |  |
| 158078 | 0.452 | 0.130 | 1.21E-14 | 88.65 | ADP binding | Cellular defense |
| 319537 | 0.122 | 0.070 | 3.88 E-8 | 88.82 | Nucleic acid binding | DNA Biosynthesis |
| 26734 | 0.172 | 0.105 | 3.88E-8 | 83.95 | Nucleic acid binding | DNA Biosynthesis |
| 162642 | 0.046 | 0.024 | 2.17E-6 | 85.6 | Retrotransposon |  |
